# Evaluation of auto-planning in VMAT for locally advanced nasopharyngeal carcinoma

**DOI:** 10.1038/s41598-022-07519-3

**Published:** 2022-03-09

**Authors:** Chen Jihong, Chen Kaiqiang, Dai Yitao, Zhang Xiuchun, Chen Yanyu, Bai Penggang

**Affiliations:** 1 Department of Radiation Oncology, Fujian Medical University Cancer Hospital, Fujian Cancer Hospital, Fuzhou, 350014 Fujian China; 2 School of Nuclear Science and Technology, University of South China, Hengyang, 421001 China

**Keywords:** Head and neck cancer, Biological physics, Radiotherapy

## Abstract

The aim of this study is to demonstrate the feasibility of a commercially available Auto-Planning module for the radiation therapy treatment planning for locally advanced nasopharyngeal carcinoma (NPC). 22 patients with locally advanced NPC were included in this study. For each patient, volumetric modulated arc therapy (VMAT) plans were generated both manually by an experienced physicist and automatically by the Auto-Planning module. The dose distribution, dosimetric parameters, monitor units and planning time were compared between automatic plans (APs) and manual plans (MPs). Meanwhile, the overall stage of disease was factored into the evaluation. The target dose coverage of APs was comparable to that of MPs. For the organs at risk (OARs) except spinal cord, the dose parameters of APs were superior to that of MPs. The D_max_ and V_50_ of brainstem were statistically lower by 1.0 Gy and 1.32% respectively, while the D_max_ of optic nerves and chiasm were also lower in the APs (p < 0.05). The APs provided a similar or superior quality to MPs in most cases, except for several patients with stage IV disease. The dose differences for most OARs were similar between the two types of plans regardless of stage while the APs provided better brainstem sparing for patients with stage III and improved the sparing of the parotid glands for stage IV patients. The total monitor units and planning time were significantly reduced in the APs. Auto-Planning is feasible for the VMAT treatment planning for locally advanced NPC.

## Introduction

Nasopharyngeal carcinoma (NPC) is one of the most common malignancies in Southeast Asia and China^1^. Radiotherapy has become the preferred treatment due to its high efficacy in the management of this disease. With recent development in technology, VMAT, as an advanced form of radiation delivery, has been widely used in the treatment of NPC^2^. Compared to intensity-modulated radiation therapy (IMRT), VMAT generally improves the target coverage and OAR sparing for head and neck tumors^3–5^. However, due to the highly complex, irregular tumor shapes and numerous radiation sensitive surrounding OARs, the radiotherapy plan design and optimization for NPC is technically challenging and resource demanding to meet strict dosimetric criteria. In order to obtain a high-quality individualized treatment plan, radiotherapy physicists spend significantly more time and effort iteratively modifying optimization functions and evaluating the results when compared to planning for other sites. Furthermore, the quality of treatment plans usually depend largely on the planners’ skill and experience, which could vary considerably among physicists and treatment centers^6,7^.

As a promising solution, automatic planning has been proposed to reduce planning time, improve quality and consistency with minimal manual intervetion^8^. Commercial softwares have been developed and introduced to clinics, including RapidPlan (Varian Medical Systems, Palo Alto, CA)^9,10^, Multi-Criteria Optimization (RaySearch Laboratories, Stockholm, Sweden)^11,12^ and Auto-Planning (Philips Medical Systems, Best, The Netherlands)^13–15^. The Auto-Planning module integrated in Pinnacle^3^ treatment planning system (TPS) could automatically place treatment isocenter, elect beam angles, contour auxiliary structures, setup optimization goals, dose constraints, and tweak weighting factors among the goals and constraints^14^. It has been demonstrated that automatic planning was feasible for many areas of treatment sites, including head and neck^13,15,16^, lung^17^, breast^18^, esophagus^7,19^, pelvic^20^ and so on. In most studies, automatic planning could achieve similar or better results compared with manual plans. However, there have been few reports on the usage of automatic planning for NPC, especially for locally advanced NPC^21^.

Due to the irregular shape of the target volumes and numerous nearby OARs, the design of treatment plans for locally advanced NPC was really time-consuming and challenging. In particular, the parotid glands and brainstem are often close to or partially overlapping with the targets. Common side effects like dry mouth^22^ and fatigue^23^ caused by radiation to the sensitive organs could seriously affect patients’ quality of life after radiotherapy. It is difficult to optimize a VMAT plan that could provide adequate target coverage while spare OARs as much as possible, even for a skillful and experienced physicist. Therefore, automatic planning for locally advanced NPC could potentially bring significant improvement in plan quality, consistency and clinical workflow efficiency. In this paper, the Auto-Planning module in Pinnacle^3^ was used to generate VMAT plans for 22 patients with locally advanced NPC. The feasibility and efficacy of Auto-Planning were evaluated by comparing dosimetry against the corresponding manual VMAT plans generated from a skilled planner. Furthermore, the difference of plan quality as a factor of the overall stages was separately analyzed for a more comprehensive evaluation.

## Material and methods

### Patient characteristics

Between October 2020 and February 2021, 22 locally advanced NPC patients who received treatment in Fujian tumor hospital were retrospectively studied. There were 17 males and 5 females aged 30 years to 76 years (median age: 48 years). The overall stage distribution was stage III: 50% (11) and IVA/B: 50% (10 IVA and 1 IVB), according to the Chinese 2008 staging system for NPC. The specific staging information was listed in Table 1. The study has been approved by the ethics committee of Fujian Cancer Hospital (ethics number: SQ2016-048-01) and all patients provided written informed consent prior to enrollment in the study. All methods were performed in accordance with the Declaration of Helsinki as well as relevant guidelines and regulations.

**Table 1:** The characteristics of patients with nasopharyngeal carcinoma (n = 22).

T stage		N stage		Overall stage	
T1	1	N0	2	Stage I	0
T2	3	N1	10	Stage II	0
T3	13	N2	3	Stage III	11
T4	5	N3	7	Stage IV	11
Total	22	Total	22	Total	22

### Target volume delineation and dose prescription

All patients were immobilized using a thermoplastic mask in the supine position. Planning CT with a slice thickness of 3-mm (Brilliance CT Big Bore, Philips Medical Systems Inc., Cleveland, OH, USA) and pretreatment enhanced magnetic resonance imaging (Philips Achieva 3.0 T) were acquired. The target volumes were contoured by experienced physicians in accordance with institutional protocols. The primary nasopharyngeal tumor (GTV-T) and definitive left and right lymph nodes (GTV-NL and GTV-NR) were determined from imaging studies, endoscopic examinations and clinical exams. A high risk region (CTV1) was defined as GTV-T with a margin of 5–10 mm, including the nasopharyngeal mucosa, while a low risk region (CTV2) was defined as potentially involved regions. The bilateral low-risk nodal regions (CTV-NL and CTV-NR) included disease at levels II–V. The seven planning target volumes (PTVs) were obtained by 3 mm uniform expansion from corresponding target volume, including GTV-T-P, CTV1-P, CTV2-P, GTV-NL-P, GTV-NR-P, CTV-NL-P and CTV-NR-P. The OARs, including lens, eyes, optic nerves, optic chiasm, brainstem, spinal cord, parotid glands, temporal lobe, mandible, temporomandibular joint, oral cavity and thyroid were also delineated and verified by the same oncologist.

### Treatment planning and dose prescription

The prescribed dose was 69.96 Gy to GTV-T-P/GTV-NL-P/GTV-NR-P, 60.06 Gy to CTV1-P, 56.1 Gy to CTV2-P and 52.8 Gy to CTV-NL-P/CTV-NR-P. Manual VMAT plans (MPs) were generated in the Pinnacle^3^ (version 16.2, Philips Radiation Oncology Systems, Madison, WI). The Auto-Planning module was used to create automatic VMAT plans (APs). Both plans were designed by the same physicist with Chinese Linear Accelerator Physicist Certificate and 7 + years experience. All plans were created for an Elekta Synergy accelerator using a pair of 6MV coplanar full arcs (178–182) with opposite 10 degree collimator rotation from their neutral position. The gantry spacing was set to 4° in each arc. Treatment goals for MPs included 100% of prescription dose to cover 95% of the target volumes and the OAR dose limitation listed in Table 2. Meanwhile, the AP template (Table 3) was used for all APs and the template parameters could be adjusted based on the patients’ anatomy. All “Ring” structures are also automatically generated. Up to three slight manual interventions were allowed in AP when deemed necessary by the planner.

**Table 2:** The criteria of OAR for manual planning.

OARs	Criteria
Left/right lens	D_max_ < 8 Gy
Left/right optic nerves	D_max_ < 54 Gy
Optic chiasm	D_max_ < 54 Gy
Brainstem	D_max_ < 60 Gy
Spinal cord	D_max_ < 45 Gy
Left/right parotid	D_mean_ < 30 Gy V_30_ < 50%
Thyroid	V_40_ < 80%

**Table 3:** OARs optimization goals in treatment planning.

OARs	Type	Dose (Gy)	Volume (%)	Priority	Compromise
Left lens	Max dose	5	–	High	No
Right lens	Max dose	5	–	High	No
Left optic nerves	Max dose	48	–	High	No
Right optic nerves	Max dose	48	–	High	No
Optic chiasm	Max DVH	48	0	High	No
Brainstem	Max DVH	50	5	High	Yes
Brainstem	Max DVH	40	15	High	Yes
Spinal cord	Max dose	38	–	High	No
Parotids left	Max DVH	40	20	Medium	Yes
Parotids left	Max DVH	26	45	Medium	No
Parotids right	Max DVH	40	20	Medium	Yes
Parotids right	Max DVH	26	45	Medium	Yes
Oral cavity	Max DVH	30	70	Low	Yes
Thyroid	Max DVH	40	60	Low	Yes
Mid	Max dose	38	–	Medium	Yes
Ring1	Max DVH	50	10	Medium	Yes
Ring1	Max dose	52	–	Medium	Yes
Ring2	Max DVH	45	8	Medium	Yes
Ring2	Max dose	47	–	Medium	Yes
Ring3	Max DVH	40	6	Medium	Yes
Ring3	Max dose	42	–	Medium	Yes
Ring4	Max DVH	35	4	High	Yes
Ring4	Max dose	37	–	High	Yes
Ring5	Max DVH	30	2	High	Yes
Ring5	Max dose	32	–	High	Yes

### Plan evaluation and statistical analysis

For quantitative comparisons, several dosimetric parameters were collected. Planning target volumes (PTVs) dose corresponding to 2% of volume (D_2_), 95% of volume (D_95_) and 98% of volume (D_98_), conformity index (CI = (V_prescription in PTV_/V_PTV_)*(V_prescription in PTV_/V_prescription_)) and homogeneity index ((HI = (D_2_ − D_98_)/D_prescription_)) were all evaluated. For parallel OARs such as parotid glands, mean dose (D_mean_) or V_x_ (the percentage volume receiving × Gy dose) were analyzed. For serial OARs such as spinal cord, D_max_ or D_2cc_ (max dose or dose corresponding to 2 cc volume) were calculated. Meanwhile, the monitor unit (MU) per fraction and planning time were also recorded for comparison.

The Wilcoxon’s signed rank test was carried out between APs and MPs for dosimetric parameters previously described. Statistical package for the Social Sciences (SPSS 21.0; SPSS Inc., Chicago, IL, USA) was used to perform these tests and p < 0.05 was considered statistically significant.

## Result

### Targets dose comparison

Most of the plans including APs and MPs met the prescribed requirement of targets, as shown in Table 4. In general, the passing rate of dose criteria and dose distribution in the targets were similar in the two groups of plans. For GTV-NL-P, GTV-NR-P, CTV1-P and CTV2-P, there are no statistical differences between APs and MPs. However, compared to the MPs, the D_2_ and HI of GTV-T-P was slightly higher in the APs by 0.7% and 2.8% (p < 0.05), indicating the existence of hotter dose volumes in the APs in the target volumes, e.g., D_95_ for CTV-NL-P and CTV-NR-P were higher in the APs.

**Table 4:** Dosimetric comparison of PTVs in manual and automatic VMAT (mean ± SD).

Targets	Index	Criteria	Pass rate (%)		Mean dese ± SD		p
			AP	MP	AP	MP	
GTV-T-P	D_2_(Gy)				75.11 ± 0.47	74.59 ± 0.61	0.016
	D_95_(Gy)	> 69.96	95.45	95.45	70.23 ± 0.37	70.29 ± 0.28	0.664
	HI				0.071 ± 0.010	0.069 ± 0.012	0.025
CTV-1-P	D_95_(Gy)	> 60.06	100.00	100.00	63.73 ± 1.12	63.89 ± 1.23	0.218
	CI				0.342 ± 0.121	0.345 ± 0.116	0.372
CTV-2-P	D_95_(Gy)	> 56.1	100.00	95.45	58.13 ± 0.81	57.59 ± 1.06	0.071
	CI				0.368 ± 0.094	0.435 ± 0.212	0.291
GTV-NL-P	D_2_(Gy)				73.99 ± 0.88	73.84 ± 1.03	0.084
	D_95_(Gy)	> 69.96	95.45	95.45	70.42 ± 0.46	70.23 ± 0.36	0.092
	HI				0.061 ± 0.017	0.060 ± 0.019	0.075
GTV-NR-P	D_2_(Gy)				74. 08 ± 0.64	73.91 ± 0.66	0.138
	D_95_(Gy)	> 69.96	95.45	90.91	70.31 ± 0.36	70.13 ± 0.34	0.073
	HI				0.067 ± 0.012	0.065 ± 0.013	0.067
CTV-NL-P	D_95_(Gy)	> 52.8	100.00	95.45	53.98 ± 1.26	53.65 ± 1.47	0.000
CTV-NR-P	D_95_(Gy)	> 52.8	95.45	95.45	54.35 ± 0.68	53.61 ± 0.49	0.000
PTV-6996	CI				0.941 ± 0.021	0.943 ± 0.016	0.638
PTV-5280	CI				0.440 ± 0.062	0.444 ± 0.067	0.848

### OARs dose comparison

The dosimetric parameters for all OARs were summarized in the Table 5. The passing rate of dose criteria for all OARs was similar or increased in the APs compared with MPs, except for V_30_ of right parotid gland. Meanwhile, most dose parameters for APs were lower than MPs. The D_max_ of left and right optic nerves, chiasm and brainstem were decreased by 1.9 Gy, 2.4 Gy, 1,2 Gy and 1.0 Gy in the APs, respectively (p < 0.05). The V_50_ of brainstem, D_2cc_ of mandible and D_mean_ of oral cavity were also statistically lower in the APs, by 1.32%, 1.0 Gy and 1.5 Gy. However, the max dose of spinal cord was increased by 1.0 Gy in the APs (p < 0.05), although such increase at dose levels around 40 Gy was clinically insignificant. In addition, the volume of the low-dose (< 30 Gy) regions was significantly decreased from 2497.8 cc in MP to 2395.6 cc (p < 0.05), indicating an improvement in dose conformity and overall better sparing in normal tissues.

**Table 5:** Dosimetric comparison of OARs in manual and automatic VMAT (mean ± SD).

OARs	Index	Criteria	Pass rate (%)		Mean dese ± SD		p-value
			AP	MP	AP	MP	
Left lens	D_max_ (Gy)	< 8 Gy	100.00	100.00	4.76 ± 1.24	4.80 ± 1.04	0.768
Right lens	D_max_ (Gy)	< 8 Gy	100.00	100.00	5.33 ± 2.19	5.01 ± 1.17	0.911
Left optic nerves	D_max_ (Gy)	< 54 Gy	86.36	81.82	38.28 ± 16.76	40.18 ± 15.95	0.016
Right optic nerves	D_max_ (Gy)	< 54 Gy	81.82	77.27	41.45 ± 16.74	43.88 ± 17.37	0.003
Optic chiasm	D_max_ (Gy)	< 54 Gy	81.82	72.73	46.92 ± 15.08	48.15 ± 13.28	0.040
Brainstem	D_max_ (Gy)	< 54 Gy	54.55	45.45	52.98 ± 7.76	53.96 ± 6.55	0.046
Brainstem	V_50_ (%)	< 5%	72.73	63.64	7.94 ± 7.67	9.26 ± 8.05	0.009
Spinal cord	D_max_ (Gy)	< 45 Gy	100.00	100.00	40.68 ± 1.52	39.65 ± 0.97	0.003
Parotid left	D_mean_ (Gy)	< 30 Gy	22.73	0.00	34.01 ± 6.13	34.56 ± 5.33	0.181
	V_30_ (%)	< 50%	81.82	81.82	46.13 ± 15.51	47.74 ± 13.17	0.149
Parotid right	D_mean_ (Gy)	< 30 Gy	18.18	4.55	35.18 ± 5.49	35.36 ± 4.10	0.205
	V_30_ (%)	< 50%	50.00	54.55	49.76 ± 14.28	49.60 ± 9.35	0.394
TM joint	D_2cc_ (Gy)	< 70 Gy	50.00	50.00	60.12 ± 10.57	61.13 ± 9.51	0.108
Mandible	D_2cc_ (Gy)	< 70 Gy	95.45	95.45	58.11 ± 7.54	59.15 ± 6.72	0.006
Temporal lobes	D_2cc_ (Gy)	< 60 Gy	77.27	77.27	60.11 ± 6.51	60.27 ± 6.69	0.455
Oral cavity	D_mean_ (Gy)	< 45 Gy	100.00	95.45	32.96 ± 4.88	34.49 ± 6.39	0.013
Thyroid	V_40_ (Gy)	< 80%	72.73	68.18	64.11 ± 26.34	65.69 ± 23.34	0.274

### MU and planning time comparison

The average MU was 643.59 ± 45.42 in APs and 672.12 ± 51.82 in MPs respectively. The APs reduced the average MU by 4.2% (p < 0.05).

The overall treatment planning time of APs, including the manual intervention time and computer calculation time, were statistically reduced by 26.3% in AP plans, from 144 to 106 mins (p < 0.05).

### Stratified analysis by the overall stage

The locally advanced NPC patients were divided into two groups, according to the overall stage. The differences in selected dosimetric characteristics between APs and MPs for the targets and OARs were calculated separately for the two groups, as shown in Table 6. The results suggested that the dose difference for the targets was independent of overall stage as the values were similar and without statistical difference (not listed in Table 6).

**Table 6:** Differences in dosimetric parameters between manual and automatic VMAT for the OARs stratified by overall stage (mean ± SD).

OARs	Index	Stage 3		Stage 4	
		Δ	p-value	Δ	p-value
Left lens	D_max_ (Gy)	− 0.04 ± 0.74	0.929	0.10 ± 0.85	0.646
Right lens	D_max_ (Gy)	0.34 ± 0.69	0.155	− 0.97 ± 1.56	0.139
Left optic nerves	D_max_ (Gy)	2.02 ± 6.92	0.091	1.79 ± 7.21	0.093
Right optic nerves	D_max_ (Gy)	1.39 ± 4.78	0.062	1.48 ± 3.39	0.017
Optic chiasm	D_max_ (Gy)	1.69 ± 3.27	0.033	0.78 ± 4.87	0.541
Brainstem	D_max_ (Gy)	1.17 ± 2.41	0.110	0.79 ± 2.23	0.285
Brainstem	V_50_ (%)	2.20 ± 2.13	0.024	0.44 ± 1.77	0.005
Spinal cord	D_max_ (Gy)	− 1.46 ± 1.83	0.028	− 0.59 ± 1.00	0.043
Parotid left	D_mean_ (Gy)	0.46 ± 1.92	0.248	0.64 ± 2.02	0.169
	V_30_ (%)	1.28 ± 5.25	0.424	1.95 ± 5.63	0.333
Parotid right	D_mean_ (Gy)	− 0.37 ± 3.97	0.657	0.74 ± 1.78	0.139
	V_30_ (%)	− 2.09 ± 12.26	0.929	1.78 ± 4.64	0.241
TM joint	D_2cc_ (Gy)	0.96 ± 2.35	0.220	1.05 ± 2.64	0.333
Mandible	D_2cc_ (Gy)	1.41 ± 1.90	0.050	0.67 ± 0.94	0.074
Temporal lobes	D_2cc_ (Gy)	0.21 ± 1.29	0.534	0.11 ± 1.12	0.646
Oral cavity	D_mean_ (Gy)	0.67 ± 2.56	0.285	2.39 ± 3.08	0.013
Thyroid	V_40_ (Gy)	4.93 ± 8.12	0.050	− 1.77 ± 6.54	0.386

In both groups, APs might result in superior dose sparing for most OARs than MPs, except spinal cord. The improvement appeared most for optic chiasm and brainstem in Stage III, and parotid glands in Stage IV. Although most differences were statistically insignificant, V_50_ of brainstem reduced more evidently for stage III in APs (2.2% vs 0.4%, p < 0.05). V_30_ and D_mean_ of parotid glands reduced more evidently for stage IV. Nevertheless, the maximum dose to the spinal cord was lower in MPs for both stages and the difference was greater in stage III (p < 0.05). However, the differences are unlikely to be clinically significant at 40 Gy levels.

## Discussion

A good amount of clinically used IMRT plans could be further optimized and improved, especially for those designed with limited time constraints, inadequate computational resources or less experienced planners^24^. Recently, automatic IMRT planning was fastly developed to potentially improve the plan quality and clinical efficiency. For example, the Auto-Planning module in the Pinnacle TPS is able to adjust optimization parameters and generate clinically acceptable plans automatically, based on an optimization algorithm with minimal planner intervention^14^. In this study, we demonstrated the feasibility and efficiency of Auto-Planning module in the VMAT planning for locally advanced NPC.

For both APs and MPs, the dose criteria of targets and OARs could not be fully met because some of the OARs were close to or overlapping with the targets. In general, the target dose coverage of APs was similar to that of MPs. It was notable that while the dose uniformity for GTV-T-P was superior in the MPs, dose inhomogeneity in tumors could be of less clinical concern in the era of imaging guided radiotherapy (IGRT) and inter fractional adaptive planning. In addition to providing preferable brainstem sparing, AP passing rates for target dose goals were equal to or slightly higher than MPs.

For most of the OARs, the dosimetric parameters of APs were superior to that of MPs, while the passing rates were usually higher than or equal to MPs, as also concluded by Yang et al.^25^ and Wang et al.^26^. APs could automatically generate a number of auxiliary structures for the dose limiting, which was practically impossible difficult to accomplish manually. However, the average D_max_ of spinal cord for MPs was 1.03 Gy lower than that of APs (p < 0.05). In this particular case, a better balance between the particular OAR dose constraints and targets might be reached by an experienced physicist repeatedly adjusting the related parameter settings^14^.

**Figure 1:**
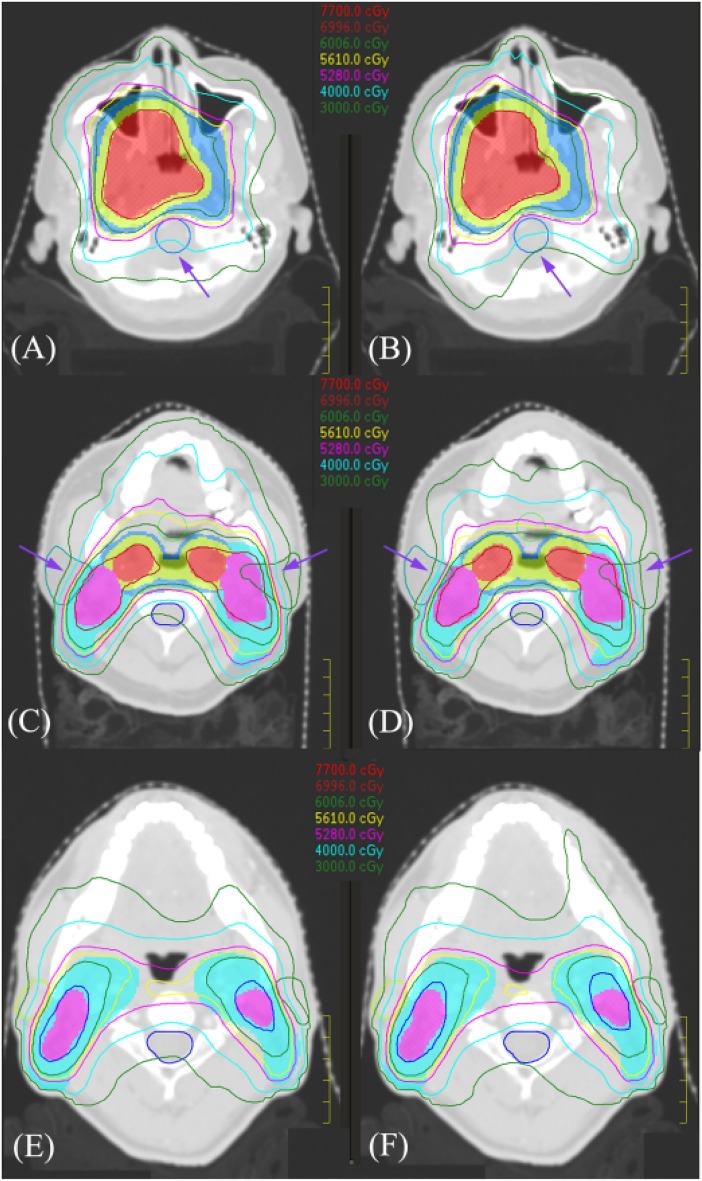
The dose distributions for two representative NPC patients were displayed. (**A**): manual plan, (**B**): automatic plan for a patient with stage III (T3N2M0); (**C**): manual plan, (**D**): automatic plan for a patient with stage IV (T4N3M0); (**E**): manual plan, (**F**): automatic plan for a patient with stage IV (T3N1M0).

For locally advanced NPC, dose differences for most OARs were similar between AP and MP plans regardless of overall stage. However, the APs provided better brainstem sparing in some stage III patients. As shown in Fig. 1A and B, there might be sufficient anatomic distance between brainstem and tumor targets in this patients’ cohort. Zhang et al. have reported that automatic plan would be more effective in sparing the brainstem if the anatomic distance between targets and the pons was greater than 5 mm^27^. In addition, the parotid glands sparing in patients with stage IV seemed superior for the automatic plan. However, when focusing on particular patients with stage IV, the parotid glands could be overprotected in the APs at the cost of reduced dose coverage for GTV-NL-P and GTV-NR-P. The parotid glands were more or less overlapping with the target in Stage IV. A typical dose distribution for a patient in this cohort was shown in Fig. 1C and D. The parotid glands were clearly better protected in the AP, but there was a significant underdose in the overlapping region between targets and parotid glands. In this case, the automatic plan could still not meet the dose criteria for parotid glands, which was usually not acceptable by the clinician. Our finding suggested that the balance between parotid glands protection and target dose coverage was still a challenge even for the Auto-Planning, especially for stage IV patients. For example, if the exposure dose of parotid glands was close to the dose criteria (V_30_ ≤ 50%), the clinician in our institution would tend to selectively reduce the CTV margins in favor of the protection of parotid glands. Conversely, if the exposure dose of parotid glands was far exceeded the dose criteria, adequate target coverage would be the preferred choice. For some other cases, the plan quality of APs was comparable to MPs. As shown in Fig. 1E and F, dose distribution of the two plans was quite similar in this patient. In our study, when specific dose distribution and dosimetric parameters were considered, the plan quality of APs was superior or equal to MPs in most cases, and inferior to MPs in several patients with stage IV.

Overall, the design of a conventional VMAT radiotherapy for locally advanced NPC could benefit from the automatic planning. In most cases, automatic plans were expected to achieve a similar or better plan quality. However, in several stage IV patients, automatic planning might have over-protected certain OARs such as the parotid glands. In such cases, it would require manual intervention from an experienced physicist and further clarifications for clinical preferences from the clinician. In addition, automatic planning could improve the planning efficiency. AP was usually based on artificial intelligence (AI) through the application of predictive models and decision supporting systems (DSS) optimization. It was particularly suitable for repetitive iterative work^28^. The overall planning time was decreased by 26% in the automatic planning, consistent with previous studies^21,29^. In fact, the improvement in planning efficiency was much greater than a flat reduction in planning time since the physicist can work on other duties while the automatic planning is being carried out by treatment planning computers backstage. This feature shall be particularly beneficial to institutions with a large number of patients but limited planners. Meanwhile, the patients who needed frequent replanning due to rapid changes in anatomy during a course of therapy could benefit from AP as the turnaround time of replan is expected to be much shorter. In this study, a uniform template parameter setting was used to start the AP process. The lack of individuality in this initialization may place unnecessary challenges to the AP algorithm in finding an optimized dose distribution with respect to a patient’s individual anatomy. Recent advancements in AI-based automatic planning are developing rapidly and potentially they could take the individual anatomy into account^30–33^. Bai et al. has developed a neural network-based IMRT treatment planning technique for locally advanced NPC^33^. Then automatic IMRT plan could be generated based on the individual’s anatomy, with comparable dosimetric qualities to manual plan^33^. However, these automatic plannings were usually difficult to be integrated into a commercial TPS platform. It demands high quality data management and computer skills among physicists. Conversely, the Auto-Planning module in its current format was more clinically adapted and easier to implement in practice.

## Conclusion

For locally advanced NPC, Auto-Planning module could generate VMAT plans with similar or superior plan quality compared to manual VMAT plans for most patients. However, manual approach could be preferred in certain stage IV patients, due to a better control of the balance between the OARs and targets by an experienced physicist. In general, automatic VMAT could greatly improve clinical efficiency and should be an option for the implementation of locally advanced NPC VMAT treatment planning after careful institutional validation.
